# Clinicopathologic Findings of Spontaneous Leukemia in 9 Pet African Hedgehogs (*Atelerix Albiventris*)

**DOI:** 10.3389/fvets.2020.00054

**Published:** 2020-02-11

**Authors:** Iori Koizumi, Daniela Hernandez-Muguiro, Shelley Ann Ash Chu, Tracy Stokol, Midori Goto Asakawa

**Affiliations:** ^1^Koizumi Nest Animal Hospital, Fukuoka, Japan; ^2^CityU Veterinary Diagnostic Laboratory, City University of Hong Kong, Hong Kong, Hong Kong; ^3^Department of Population Medicine and Diagnostic Sciences, College of Veterinary Medicine, Cornell University, Ithaca, NY, United States; ^4^Veterinary Specialists Emergency Center, Saitama, Japan

**Keywords:** cancer, acute leukemia, lymphoma, histopathology, cytology, immunohistochemistry, eosinophilic leukemia

## Abstract

Previous reports of leukemia in hedgehogs are limited. We describe clinicopathologic features of leukemia in 9 hedgehogs, including eosinophilic leukemia (*n* = 3) and acute leukemia/leukemic phase of lymphoma (*n* = 6). All 3 hedgehogs with eosinophilic leukemia were older than 2 years of age; in contrast, 4 of 6 cases of acute leukemia/lymphoma were <2 years old. Hedgehogs presented for non-specific clinical signs of anorexia and lethargy. On hematologic testing, hedgehogs with eosinophilic leukemia had a marked leukocytosis, consisting mostly of eosinophilic precursors with fewer mature eosinophils, whereas there were 43–97% immature cells (blasts) in the blood of hedgehogs with acute leukemia/lymphoma. Anemia (*n* = 6) and/or thrombocytopenia (*n* = 6) were concurrent findings. Increased liver enzyme activities (alanine aminotransferase, alkaline phosphatase) and hypoalbuminemia were the common findings on biochemical panels. All cases of eosinophilic leukemia and 4 cases of acute leukemia/lymphoma died shortly after diagnosis (median 7 days, range 0–41 days), whereas 2 cases of acute leukemia/lymphoma lived for 94 or 101 days. Postmortem examination in 5 cases (1 eosinophilic leukemia, 4 acute leukemia/lymphoma) showed bone marrow infiltrates, confirming eosinophilic leukemia and acute leukemia in 1 and 3 cases, and bone marrow necrosis in 1 animal with acute leukemia/lymphoma. Immunohistochemical staining of bone marrow sections confirmed a T-cell acute leukemia in 1 case. Several hedgehogs had concurrent carcinomas. Hedgehogs suffer from eosinophilic leukemia and acute leukemia/lymphoma. However, classification of acute leukemia by lineage was not possible due to lack of hedgehog cross-reactive or species-specific reagents.

## Background

Spontaneous neoplasia is common in African Pygmy hedgehogs, with a reported disease prevalence of 30–60% (1–6). The median age of affected animals ranges from 2.5 to 3.5 years, with most pet hedgehogs only living for 3 to 6 years (1, 5, 7). Tumors most commonly arise from the skin, lymphoid tissue, and gastrointestinal, endocrine, and reproductive systems (1, 2, 5, 6) and are usually malignant (up to 85%) (1). Lymphoma is the most common reported type of hematopoietic tumor, with a disease prevalence ranging from 2 to 9% (1, 5, 6). In one study of 63 cases, lymphoma was the second most common reported tumor (6). In these reports, lymphoma usually involved multicentric peripheral lymph nodes, (1) with a few case reports of gastrointestinal, cutaneous and splenic forms (8–11).

Leukemia is defined as a hematopoietic neoplasm involving the blood and/or bone marrow. Leukemia can be further classified as acute or chronic, based on the degree of cellular differentiation, and as myeloid, lymphoid, or mixed lineage, based on the cell lineage. Acute leukemias which lack detectable differentiation markers are classified as acute undifferentiated leukemia (12). With lymphoid neoplasms, there is substantial overlap between primary leukemia and lymphoma, the latter of which can have a leukemic phase (tumor cells in circulation and/or bone marrow). For this reason, certain lymphoid neoplasms are grouped into the categories of precursor or immature leukemia/lymphoma or mature leukemia/lymphoma by the World Health Organization (12). Leukemia is rare in hedgehogs, with 2 affected animals with suspect myeloid leukemia in one case series of 36 hedgehogs (1) and briefly mentioned cases of presumptive myeloid leukemia in retrospective studies of spontaneous tumors in exotic mammals (13–16). In these reports, the myeloid leukemia was classified as eosinophilic leukemia or was not specified (1, 13–16). There has also been one case of a hedgehog with gastrointestinal lymphoma and a marked lymphocytosis (48,000/μL), suggesting a leukemic phase of lymphoma (11). This report aims to describe the historical, clinicopathologic, and postmortem findings of various types of leukemia in 9 hedgehogs, including 3 cases of eosinophilic leukemia and 6 cases of acute leukemia or intermediate to large cell lymphoma (acute leukemia/lymphoma). The cases were diagnosed at two hospitals in Japan (Veterinary Specialists and Emergency Center, Saitama; and Koizumi Nest Animal Hospital, Fukuoka) and one academic institution in the USA (Cornell University, Ithaca, NY) between 2013 and 2018. All but one cases were privately owned animals in Japan.

## Case Presentation

### Eosinophilic Leukemia

**Figure 1:**
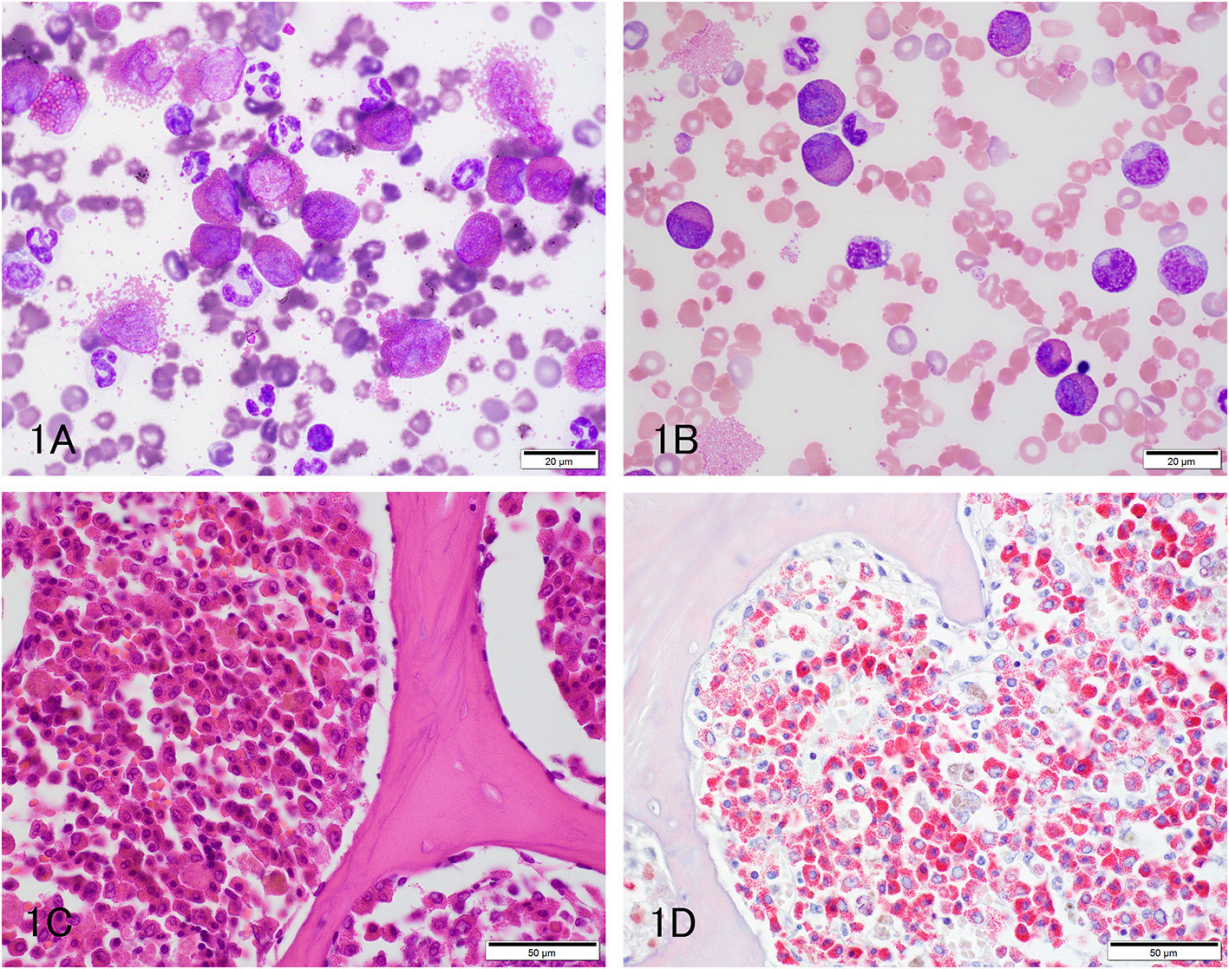
Eosinophilic leukemia in pet African Hedgehogs. **(A,B)** Representative photomicrographs of a blood smear from 2 hedgehogs with eosinophilic leukemia (**A**: Case 1; **B**: Case 2). Immature eosinophils dominate, with fewer mature eosinophils and neutrophils (**A**: Diff-quik; **B**: Modified Wright's stain). There are numerous polychromatophils in the smear from case 2, supporting a regenerative response to the anemia. Both animals were thrombocytopenic. **(C)** Representative photomicrograph of a formalin-fixed section of femoral bone marrow from a hedgehog with eosinophilic leukemia (case 3). The marrow was replaced by sheets of eosinophils, which were mostly immature (myelocytes or metamyelocytes) with few other hematopoietic cells (hematoxylin & eosin stain). **(D)** Representative photomicrograph of a formalin-fixed section of femoral bone marrow from a hedgehog with eosinophilic leukemia (case 3). The eosinophil granules stain prominently with Luna stain (Luna stain).

Three hedgehogs were diagnosed with eosinophilic leukemia (cases 1 to 3). Animals were between 2 and 4 years of age and a mix of sexes (Table 1). They were presented for anorexia (*n* = 2), lethargy (*n* = 2) and collapse (*n* = 1). Physical and imaging findings were variable, with one animal having an osteolytic gingival mass and a second animal having hepatosplenomegaly (Table 1). A hemogram with a manual differential cell count revealed moderate to marked leukocytosis (estimated or measured leukocyte count) due primarily to an eosinophilia (Table 2). Eosinophils were mostly immature and dominated by metamyelocytes or myelocytes (91–100%) with low percentages of band or segmented eosinophils, Figures 1A,B). All affected animals had a concurrent neutrophilic leukocytosis with a left shift, which was substantial in case 2, and a mild lymphocytosis. Low numbers of putative immature cells (blasts) were identified in case 2 and were large cells (15–17 μm) with fine chromatin and variably distinct nucleoli. Two animals had a mild to moderate anemia, which appeared regenerative in case 2 based on moderate to many polychromatophils in the blood smear (Figure 1B). Moderate acanthocytes with a few keratocytes and schistocytes were seen in the blood smear from case 2, indicating red blood cell fragmentation. All animals were thrombocytopenic (<150,000/uL). Biochemical abnormalities were increased alanine aminotransferase (ALT) and alkaline phosphatase (ALP) activities (*n* = 3), hyperbilirubinemia (*n* = 2), hypoalbuminemia with hyperglobulinemia (*n* = 2), and hypercalcemia (*n* = 1). Bone marrow aspirates were not performed in any case. Cytochemical staining for myeloperoxidase (MPO) (Sigma-Aldrich, St. Louis, MO, USA, procedure No. 390), ALP (Sigma-Aldrich. procedure No. 85), α-naphthyl-butyrate esterase (ANBE) (Sigma-Aldrich, procedure No. 181), chloroacetate esterase (CAE) (Sigma-Aldrich, procedure No. 91), and Sudan Black B (SBB) (Sigma-Aldrich, procedure No. 380) was performed on blood smears from case 2 and a control hedgehog with normal hemogram results. Luna staining was also done on smears from case 2 and another hedgehog with normal hemogram results. Eosinophils were positive for MPO, ALP, SBB, and Luna stain in blood smears from the control and leukemic hedgehogs. Mature eosinophils in the control and leukemic hedgehogs were negative for ANBE, but a few immature eosinophils in case 2 with eosinophilic leukemia were positive for this stain. Mature and immature eosinophils in case 2 with eosinophilic leukemia were negative for CAE, contrasting with mature eosinophils in the control hedgehog, which were positive for CAE. Neutrophils in the control and leukemic hedgehogs were also positive for CAE, serving as an internal positive control. The different staining patterns for ANBE and CAE in eosinophils of case 2 with eosinophilic leukemia were considered aberrant. The presumptive blasts were negative for all stains. Follow-up information was available on 2 hedgehogs. Case 1 died on the day of admission, whereas case 3 was treated with prednisolone (1 mg/kg SID, PO) but died 41 days later (Table 1). A postmortem examination in case 3 revealed infiltrates of eosinophilic precursors in multiple organs, including the bone marrow, liver, pancreas, kidney, lungs, lymph nodes, gastrointestinal tract, and genitourinary tract. Eosinophil precursors comprised >80% of the marrow in multiple bone sites (Figure 1C) and their granules were positive on Luna staining (Figure 1D). Neoplastic infiltrates were noted within lobules, periportal and centrilobular regions of the liver, red pulp of the spleen, interstitium of the kidney, pancreas, testicle, and prostate and submucosa of the urinary bladder, oral cavity, gastrointestinal tract, and trachea. The gingival mass was a squamous cell carcinoma with no evidence of metastasis.

**Table 1:** Clinical, physical examination and imaging findings, along with outcome and post-mortem examination in 9 hedgehogs with eosinophilic leukemia (*n* = 3) or acute leukemia/lymphoma (*n* = 6).

**Case**	**1**	**2**	**3**	**4**	**5**	**6**	**7**	**8**	**9**
Diagnosis	EL	EL	EL	ALL (T cell)	AL	AL	AL/LY	LY	AL
Age (years)	2.6	3.5	3	1.6	3	3	1.6	1.3	1.8
Body weight (g)	624	304	393	658	350	536	495	338	274
Sex	F	M	M	F	F	F	F	F	F
Clinical signs	Anorexia, lethargy, collapse	Anorexia, lethargy	None	Anorexia, dyspnea	Abdominal distension	Anorexia	Vaginal bleeding	Anorexia, acute lethargy	Anorexia, lethargy, hematuria
Physical examination and imaging findings	Hypotension, hypothermia, icterus, hepatosplenomegaly	Tachycardia, tachypnea, pallor	Gingival mass with osteolysis	Pale mucous membranes, marked splenomegaly, cervical mass	Peritoneal effusion, ovarian mass, cervical mass	Gingivitis	Marked splenomegaly, uterine mass	Gastric foreign body, mesenteric lymphadenopathy	Nasal discharge, dyspnea, gingivitis, uterine mass
Outcome	Died on admission	Unknown	Died day 41	Died day 1	Died day 94	Died day 34	Died day 9	Died day 5	Euthanized day 101
Post-mortem findings	ND	ND	Disseminated eosinophilic leukemia, squamous cell carcinoma (gingival mass)	Acute T cell leukemia, multiorgan infiltrates, splenic extramedullary hematopoiesis, thyroid carcinoma, pulmonary abscess	Disseminated carcinomatosis, extensive bone marrow infiltrates, marked splenic extramedullary hematopoiesis	Bone marrow necrosis, tumor infiltrates in liver, kidney, adrenal and stomach, hepatic lipidosis, splenic extramedullary hematopoiesis	ND	ND	Extensive bone marrow infiltrates and infiltrates in other organs
Special stains on post-mortem tissue	ND	ND	Bone marrow: Eosinophils: Luna stain(+)	ND	ND	ND	ND	ND	ND
Immunohistochemical stains on post-mortem tissue	ND	ND	ND	Bone marrow: CD3 (+) CD20 (–) Iba-1 (–)	Bone marrow: CD3 (–) CD20 (–) Iba-1 (–)	ND	ND	ND	Bone marrow: CD3 (–) CD20 (–) Iba-1 (–)

*EL, Eosinophilic leukemia; T-ALL, Acute T-cell leukemia; AL, Acute leukemia; AL/LY, Acute leukemia/lymphoma; LY, Lymphoma; F, Female; M, Male; ND, Not done*.

**Table 2:** Hematologic, bone marrow, and biochemical panel findings in 9 hedgehogs with eosinophilic leukemia (*n* = 3) or acute leukemia/lymphoma (*n* = 6).

**Case**		**1**	**2**	**3**	**4**	**5**	**6**	**7**	**8**	**9**		**Reference interval^†^**
Diagnosis		EL	EL	EL	T-ALL	AL	AL	AL/LY	LY	AL		
										Day1	Day101	
Hematology tests	Units											
Packed cell volume	%	35	23^*^	33	14	29	19	17	11	25	21	34–47
Leukocyte count	×10^3^/μL	>60	109	>30	26.9^***^	53.7	>60	>60	>60	>30	>30	11.5–21.7
Segmented neutrophils	×10^3^/μL	-	32.7	-	0.0	10.2	-	-	-	-	-	6.1–14.6
	%	23	30	30	0	19	10	0	19	3	31	
Band neutrophils	×10^3^/μL	-	22.9	-	0.0	1.6	-	-	-	-	-	
	%	2	21	1	0	3	8	0	3	0	1	
Lymphocytes	×10^3^/μL	-	9.8	-	3.7	0	-	-	-	-	-	3.3–8.9
	%	9	9	15	14	0	0	3	35	0	0	
Monocytes	×10^3^/μL	-	1.1	-	0.5	1.1	-	-	-	-	-	0–0.8
	%	7	1	5	2	2	0	0	0	3	0	
Eosinophils	×10^3^/μL	-	39.3	-	0.5	1.1	-	-	-	-	-	0–0.3
	%	59	36	47	2	2	0	0	0	0	0	
Basophils	×10^3^/μL	-	1.1	-	0.0	1.1	-	-	-	-	-	0–0.2
	%	0	1	2	0	0	0	0	0	0	0	
Other^**^	×10^3^/μL	-	2.2	-	22.1	36.5	-	-	-	-	-	NA
	%	0	2	0	82	68	82	97	43	94	68	
Nucleated RBC (nRBC)	/100 WBC	1	1	0	42	0	0	0	4	16	40	NA
Platelet count	×10^3^/μL	105	<30	131	20	258	19	36	580	129	32	NA
Platelet estimate		Low	Very low	Low	Very low	Adequate	Very low	Increased	Increased	Adequate	Low	
Macroplatelets		-	+	-	-	+	+	+	+	+	+	
Bone marrow and special stains												
Bone marrow aspirate results		ND	ND	ND	39% blasts	ND	Unsuccessful	ND	16% blasts	Unsuccessful		
Other stains		ND	Venous blood: Blasts: (–) Eosinophils: MPO (+), ALP (+) SBB (+), ANBE (+), CAE (–), Luna stain (+)	ND	Venous blood: MPO (–)	Venous blood: MPO (–)	ND	ND	ND	ND		
Biochemical tests												
Sodium	mEq/L	ND	141	133	145	ND	136	135	124	139	134	NA
Potassium	mEq/L	ND	4.9	6.1	4.3	ND	6.3	5.7	3.8	5.1	4.9	NA
Chloride	mEq/L	ND	104	ND	105	ND	ND	ND	ND	ND	ND	NA
Urea nitrogen	mg/dL	68	52	36	69	42	22	24	41	36	74	34–57
Creatinine	mg/dL	ND	1.5	0.4	0.3	0.6	0.4	0.5	0.5	0.4	0.4	0.5–1.0
Calcium	mg/dL	10.2	11.1	12.7	9.2	ND	10.9	10.4	6.9	10.1	12.1	8.6–11.4
Phosphate	mg/dL	13.8	4.7	2.9	2.6	ND	3.6	4.4	4.8	4.2	4	NA
Glucose	mg/dL	82	78	94	106	93	132	79	101	109	111	60–125
Total protein	g/dL	6	5.4	6.8	7	5.6	6.7	5.2	4.1	5.9	5.7	4.6–6.9
Albumin	g/dL	2	2.7	2.5	4.2	3.1	3.2	2.3	1.8	1.3	1.6	2.7–3.9
Globulins	g/dL	4	2.7	4.3	2.8	2.5	3.5	3.3	2.3	4.6	4.1	1.9–3.6
ALT	IU/L	196	269	54	22	60	44	40	28	41	45	15-29
ALP	IU/L	2920	795	113	272	220	34	41	103	11	12	18–26
Total bilirubin	mg/dL	3.4	3.2	0.3	0.2	ND	0.2	0.2	0.3	0.3	0.3	0.5 ± 0.1^Ψ^
Ammonia	μg/dL	420	ND	66	84	202	80	ND	105	ND	ND	NA

*EL, Eosinophilic leukemia; T-ALL, Acute T-cell leukemia; AL, Acute leukemia; AL/LY, Acute leukemia/lymphoma; LY, Lymphoma; MPO, Myeloperoxidase; SBB, Sudan Black B; ANBE, alpha naphthyl butyrate esterase; CAE, Chloroacetate esterase; ALT, Alanine aminotransferase; ALP, Alkaline phosphatase; ND, Not done; NA, Not available*.

* *Calculated hematocrit from the ADVIA automated analyzer*.

** *Other WBC are neoplastic mononuclear cells with fine to slightly clumped chromatin and variably distinct nucleoli (suspect blasts)*.

*** *WBC count was corrected for nRBC*.

† *Okorie-Kanu et al. (17)*.

Ψ *Physiological Data Reference Values, International Species Information System, 1996*.

### Acute Leukemia or Intermediate to Large Cell Lymphoma

**Figure 2:**
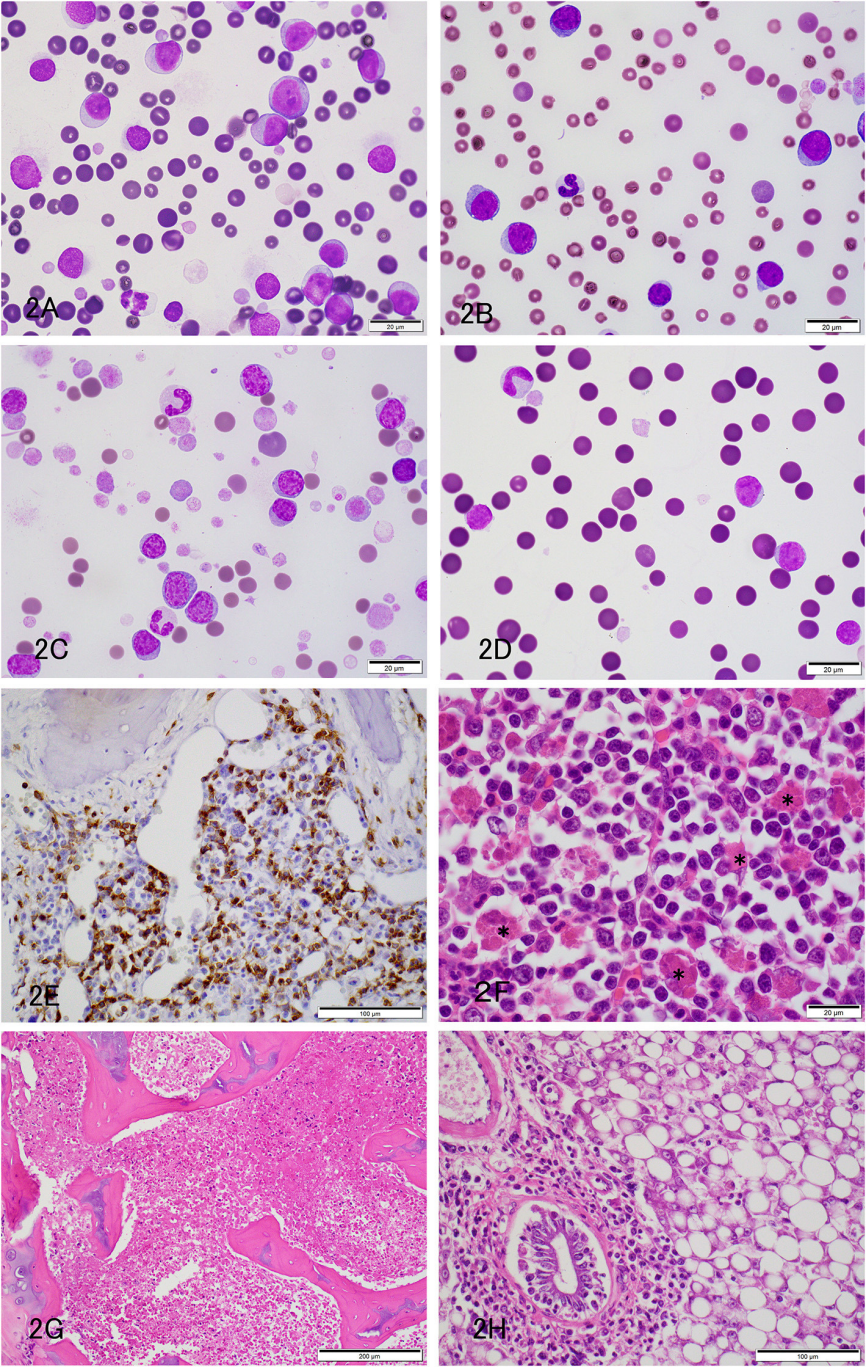
Acute leukemia or leukemia/lymphoma in pet African Hedgehogs. **(A–C)** Representative photomicrographs of blood smears from hedgehogs with acute leukemia (**A**: Case 5; **B**: Case 6) and leukemia/lymphoma (**C**: Case 7). Large round cells (12–15 μm) with fine chromatin and nucleoli dominate in cases 5 and 6, whereas intermediate to large cells with slightly clumped chromatin and inapparent nucleoli are the most numerous cells in case 7. Cases 5 and 7 also had numerous large platelets, and case 6 was thrombocytopenic. A few polychromatophils were noted in case 7 (**A,B**: Diff-quik stain, **C**: Modified Wright's stain). **(D)** Representative photomicrograph of a blood smear from a hedgehog with acute leukemia (Case 9) on the day of admission (day 1). Diff quick stain. The neoplastic cells were comprised of intermediate cells (10–12 μm), which had round nuclei with fine chromatin. **(E)** Representative photomicrograph of a formalin-fixed paraffin-embedded section of bone marrow from a hedgehog with T cell acute leukemia stained with a polyclonal rabbit antibody against CD3 (Dako) (Case 4). A diffuse infiltrate of intermediate round CD3-positive cells, comprising ~40% of cells, is seen in the bone marrow. **(F)** Representative photomicrograph of a formalin-fixed section of femoral bone marrow from a hedgehog with acute leukemia (case 5). The marrow was largely effaced by an infiltrate of neoplastic round cells. There was also a moderate histiocytosis, with macrophages phagocytizing erythrocytes (^*^) (hematoxylin & eosin stain). **(G)** Representative photomicrograph of a formalin-fixed section of femoral bone marrow from a hedgehog with acute leukemia (case 6). The marrow was diffusely necrotic (presumably tumor cells) (hematoxylin & eosin stain). **(H)** Representative photomicrograph of a formalin-fixed section of liver from a hedgehog with acute leukemia and bone marrow necrosis (case 6). Infiltrates of intermediate to large tumor cells are located in the periportal and sinusoidal regions. There is concurrent marked hepatocellular vacuolation, compatible with lipid accumulation (macrovesicular steatosis) (hematoxylin & eosin stain).

Six hedgehogs were diagnosed with acute leukemia/lymphoma (cases 4 to 9). Their median age was 1.7 years (range 1.3–3 years) and all were intact females. Clinical signs were variable and non-specific (Table 1). Physical examination and diagnostic imaging revealed splenomegaly (*n* = 2), mesenteric lymphadenopathy (*n* = 1), masses in the cervical region (*n* = 2), uterus (*n* = 2) and ovary (*n* = 1, with a concurrent peritoneal effusion), gingivitis (*n* = 2) and a gastric foreign body (*n* = 1) (Table 1). All 6 cases had a moderate to marked leukocytosis. Five of 6 cases had numerous intermediate to large (12–15 μm) neoplastic cells (blasts), comprising between 43 and 97% of the leukocytes. The blasts had round to oval nuclei with fine to slightly clumped chromatin and one to three prominent nucleoli, and small amounts of light to mid-blue cytoplasm (Figures 2A–C). The remaining case (case 9) had 94% intermediate (10–12 μm) neoplastic cells with round nuclei containing fine chromatin and indistinct nucleoli, morphologically resembling lymphocytes (Figure 2D). All animals were anemic, which was moderate to severe in 5 animals, with no or few polychromatophils (presumptive, non-regenerative) in four cases (case 4, 6–8). Case 5 had mild anemia with mildly increased polychromatophils (presumptive, regenerative), likely due to hemorrhage in ovarian carcinoma. Two animals had high numbers of circulating nucleated red blood cells (nRBC; cases 4 and 9). Although one of the latter animals did have moderate to marked polychromasia, indicating a regenerative anemia, which was attributed to abdominal hemorrhage (case 9), the nRBCs in both cases could reflect an abnormal bone marrow environment, bone marrow injury or altered splenic function. One animal was neutropenic and thrombocytopenic and another animal was thrombocytopenic at admission. Large platelets were noted in 5 animals, regardless of the count. Consistent biochemical changes were increased ALT (*n* = 4) and ALP (*n* = 5) activities (Table 2). One animal had mild hypercalcemia; however, ionized calcium was not measured. Limited cytochemical staining on blood smears from 2 animals did not help identify blast lineage (Table 2). Bone marrow aspiration was successful in 2 of 4 animals. In case 4, marrow smears were of low cellularity, with only a few marrow spicules. Rare megakaryocytes were seen. Erythroid and myeloid maturation appeared complete, however there was a decreased M:E ratio (0.07) due to a marked myeloid hypoplasia. The erythroid lineage was also considered hypoplastic due to the low marrow cellularity. There was concurrent erythroid dysplasia, characterized by binucleation, nuclear cytoplasmic asynchrony, and nuclear blebbing. Blasts comprised 39% of marrow cells. Low numbers of small lymphocytes (3.3%) and well-differentiated plasma cells (0.8%) were also noted. In case 8, the marrow was hypercellular, owing to an infiltrate of small lymphocytes (54%) and blasts (16%), as seen in blood. All other lineages were identified with normal maturation sequence and the myeloid to erythroid ratio was 1.4:1. All animals were treated with prednisolone (1–2 mg/kg once daily by mouth, subcutaneously or intravenously). Four animals died within 1 to 34 days, while the remaining two animals lived 94 and 101 days. A postmortem examination was performed in 4 animals that died between 1 and 101 days (cases 4, 5, 6 and 9). Two had concurrent epithelial tumors, including a non-metastatic thyroid carcinoma (case 4) and disseminated carcinomatosis (including the cervical mass), presumably from an ovarian carcinoma, in case 5 (Table 1). No hematopoietic tumor infiltrates were detected in extramedullary tissues and a clear infiltrate was not detected in hematoxylin-and-eosin-stained smears of bone marrow in case 4, which had 39% blasts in the blood smear. Immunohistochemical (IHC) staining of bone marrow sections for CD3 (Dako, rabbit polyclonal, USA, 1:200), CD20 (Thermoscientific, rabbit polyclonal, 1:400, UK) and Iba-1 (Wako, rabbit polyclonal, 1:200, Japan) was performed, using a histologic section of Peyer's patch from a non-leukemic hedgehog as a control. The IHC staining of the Peyer's patch in the control hedgehog showed expected positive reactions for CD3 and CD20 in lymphoid cells in the interfollicular zone and germinal center and marginal zone, respectively, and for Iba-1 in interfollicular cells with dendritic features (Supplemental Figure 1). These findings support some degree of cross-reactivity of these antibodies for hedgehog antigens. In case 4, IHC staining revealed a diffuse infiltrate of intermediate CD3-positive cells (40% of marrow cells, Figure 2E). A diagnosis of acute T cell leukemia was made in this animal, based on the lack of extramedullary infiltrates compatible with lymphoma. In case 5, there was a marked diffuse infiltrate of large round cells in the bone marrow (Figure 2F) with no evidence of extramedullary infiltrates. The tumor cells in the bone marrow were negative for CD3, CD20 and Iba-1 on IHC staining (Table 1). The final diagnosis in case 5 was acute leukemia, which could not be typed further. In case 6, there was severe diffuse marrow necrosis, presumably necrotic tumor cells (Figure 2G), but infiltrates of low numbers of large round cells were seen in the periportal region and sinusoids of the liver (Figure 2H), which had marked lipidosis. Tumor infiltrates were also evident in the interstitium of the kidney, adrenal gland, and stomach. There was extramedullary hematopoiesis in the spleen. There were too few tumor cells in these extramedullary sites to perform IHC staining. Based on the pattern of organ infiltration, a diagnosis of acute leukemia with bone marrow necrosis was made, but the leukemia could not be phenotyped further. In the longest living case (case 9), the uterine mass was surgically removed and diagnosed as a polyp on histopathologic examination. The animal was discharged with no treatment and represented 98 days later due to hematuria from a bacterial cystitis. A repeat hemogram showed persistence of the leukocytosis with neoplastic cells dominating and a concurrent neutrophilia. A persistent anemia with higher numbers of nRBCs was noted (Table 2). Biochemical results were largely unchanged, except for higher urea nitrogen and calcium concentrations (Table 2). An aspirate of a splenic mass (1.2 × 1.4 cm) identified on abdominal ultrasonography revealed mild to moderate numbers of small to intermediate cells, resembling those seen in blood. The owner elected euthanasia and postmortem examination revealed multifocal to coalescing infiltrates of medium-sized neoplastic round cells in the bone marrow, splenic red pulp, periportal and centrilobular regions of the liver, cortex of lymph nodes, and the interstitium of the renal cortex, esophagus and lungs. The neoplastic cells were negative for CD3, CD20, and Iba-1 on IHC staining of the bone marrow. A final diagnosis of acute leukemia, that could not be phenotyped further, was made. For the remaining two animals, in which a postmortem examination was not performed, the presumptive diagnosis was acute leukemia or intermediate to large cell lymphoma in case 7 (which had marked splenomegaly) and of intermediate to large cell lymphoma in case 8, which had insufficient blasts in a bone marrow aspirate to confirm an acute leukemia.

## Discussion

There are limited reports of leukemia in pet African hedgehogs and most previously reported cases were diagnosed at postmortem examination, with little description of clinical pathologic findings (13–16). Here we provide the largest case series of leukemia in these exotic species, with information on history, clinical signs, clinicopathologic and histopathologic results, and clinical outcome. The diagnosed cases were a mixture of eosinophilic leukemia and acute leukemia/lymphoma, with 1 confirmed case of T-cell acute leukemia.

The hedgehogs presented with non-specific clinical signs and were mostly intact female (78%, 7/9) and middle-aged to older animals. Hemogram results were indicative of a leukemia, revealing a marked leukocytosis, consisting mostly of immature eosinophils in animals with eosinophilic leukemia or a high proportion of blasts in animals with acute leukemia/lymphoma. Most animals had concurrent cytopenias, predominantly a non-regenerative anemia and/or thrombocytopenia, which could be due to marrow infiltrates (confirmed in 5 animals), bone marrow necrosis (seen in 1 animal, presumably necrotic tumor cells), an abnormal marrow microenvironment and concurrent peripheral causes (e.g., fragmentation hemolytic anemia, platelet consumption). Biochemical results revealed liver injury and hypoalbuminemia in most animals. The liver injury was attributed to tumor infiltrates and/or hypoxic injury whereas the hypoalbuminemia may be due to a negative acute phase response, anorexia or protein loss.

Three of the 9 cases were diagnosed as eosinophilic leukemia, based on a marked eosinophilia consisting mostly of immature cells, showing eosinophilic differentiation. Based on the lack of blasts in blood, the leukemia appeared chronic vs. acute, however bone marrow aspirates were not performed in all animals to assess the percentage of blasts. In veterinary medicine, chronic eosinophilic leukemia is a rare hematopoietic neoplasm with an indolent course that has been reported in cats (18, 19). Given the relatively high incidence in our case series and several other reports of this neoplasm in hedgehogs (15, 16), hedgehogs may have a unique genetic susceptibility to this type of leukemia. For instance, mutations in the platelet-derived growth factor receptor have been identified in humans with eosinophilic variants of leukemia (20). Eosinophilic leukemia must be differentiated from idiopathic hypereosinophilic syndrome, which can be challenging (21). In our cases, the distinction was based on the high proportion of immature eosinophils, whereas hypereosinophilic syndrome is comprised primarily of mature eosinophils. The leukemia may be more aggressive in hedgehogs because most affected animals died shortly after the diagnosis.

Four cases were diagnosed with acute leukemia based on the presence of many blasts in blood and substantial bone marrow involvement in aspirates or postmortem tissue sections without clinical, imaging or histopathologic evidence of major organomegaly or extramedullary masses. One of these cases had diffuse marrow necrosis, presumably from death of the tumor cells in marrow, since postmortem examination showed a leukemic pattern of distribution in extramedullary tissues with no obvious masses. Spontaneous bone marrow necrosis has been rarely described in acute leukemia in humans (22) and animals (23). In one case (case 7), a distinction between leukemia and lymphoma could not be made. In human patients, this distinction is typically based on the percentage of marrow blasts per WHO criteria (>25% marrow blasts is called leukemia) (12). However, since criteria have not been established in non-human animals, we based this distinction on the “main site” of involvement, being primarily blood and/or bone marrow involvement for leukemia or primarily solid tissue involvement for lymphoma. This distinction could not be done in case 7 because bone marrow was not available pre- or postmortem. Marked splenomegaly (of unknown cause) was observed clinically in this case. Another animal with acute T cell leukemia (case 4) had marked splenomegaly due to extramedullary hematopoiesis, which is a common incidental finding in laboratory rodents and has been previously reported in hedgehogs without leukemia (3). Thus, it is possible that the splenomegaly in case 7 in which a postmortem was not performed was due to extramedullary hematopoiesis vs. tumor infiltrates. In case 8, in which a bone marrow aspirate was performed, the percentage of blasts in smears of the aspirate did not fulfill criteria for an acute leukemia, either myeloid (>20%) or lymphoid (>25%) (12), and a diagnosis of lymphoma with a leukemic phase was made. There was a concurrent small cell lymphocytosis, attributed to a reactive response or tumor-infiltrating lymphocytes. However, it is possible the cells morphologically identified as small lymphocytes were part of the tumor population or that a higher percentage of blasts were present elsewhere in the bone marrow. In addition, a primary extramedullary tumor was not identified on physical or imaging findings and it is certainly possible that this hedgehog had an acute leukemia. Survival after diagnosis in the affected hedgehogs in this leukemia category was short (1 to 34 days), with two animals living for longer (94 to 101 days).

Six animals in this report were given prednisolone at 1–2 mg/kg SID, including 1 with eosinophilic leukemia and 5 with acute leukemia/lymphoma. However, the treatment was of unclear efficacy as most animals died within a month of diagnosis. The cause of death was unclear and could have been due to the leukemia or concurrent neoplasms, which were evident in 2 of the animals on postmortem examination. Our results are similar to that of other reports, where prednisone and cytarabine treatment of eosinophilic leukemia resulted in a limited response (15, 16). Given the apparent lack of response to therapy, future cases may require more aggressive chemotherapy. All but one of the hedgehogs in our case series presented to a single private hospital in Japan and genetic and geographic factors might have contributed, however, further studies would be needed to clarify the pathogenesis of hedgehog leukemia.

A limitation of this study is that the leukemia could not be phenotyped in most cases, except for the eosinophilic leukemia, where cell lineage was discernible by morphologic features alone. There are limited reagents for immunophenotyping leukemia in exotic pets or animals, such as the hedgehogs in this case series. We used antibodies against CD3 (T cell marker), CD20 (B cell marker), and Iba-1 (macrophage marker), however a positive reaction was only obtained for CD3 in one case. Cytochemical staining was done on one case of eosinophilic leukemia, along with a hedgehog control, and revealed some different staining reactions vs. the control in eosinophils, further support for a neoplastic process. However, low numbers of blasts in that animal were negative for all stains and the standard staining pattern of normal or neoplastic precursors in this species is not known. Definitive classification of leukemias in hedgehogs will require further testing and validation of different antibodies for cross-reactivity and more extensive characterization of cytochemical staining patterns in mature and immature hematopoietic cells.

## Data Availability Statement

All datasets generated for this study are included in the article/Supplementary Material.

## Ethics Statement

Ethical review and approval was not required for the animal study because the diagnosis of leukemia/lymphoma was made through routine blood examinations in veterinary clinics with owners requests and agreement. All clinicopathologic examinations were requested and agreed by the owners to obtain accurate diagnosis and to select adequate treatment for individual animals. No experimental manipulation with potential harm to animals was performed. Written informed consent was obtained from the owners for the participation of their animals in this study.

## Author Contributions

IK contributed to the clinical interpretations and description of cases in Japan. SC, DH-M, and TS contributed one case from the USA, performed the cytochemical staining and provided case details, images and description of results. MA was responsible for cytologic and histopathologic interpretation and was the lead author on the manuscript. All authors read and edited the manuscript.

### Conflict of Interest

The authors declare that the research was conducted in the absence of any commercial or financial relationships that could be construed as a potential conflict of interest.
