# Supplementary material for: Clinicopathologic Findings of Spontaneous Leukemia in 9 Pet African Hedgehogs (Atelerix Albiventris)
Source: Front Vet Sci. 2020 Feb 11;7:54. doi: 10.3389/fvets.2020.00054 (PMC7026476; doi:10.3389/fvets.2020.00054)

Supplemental Figure 1. Immunohistochemical staining on a histologic section of Peyer's patch from a non-leukemic hedgehog (control). **A:** Lymphocytes in the interfollicular are positive for CD3 (Dako, rabbit polyclonal, USA, 1:200), x40 objective, Inset: Cropped magnified image. **B:** Germinal center and marginal zone cells showed expected positivity for CD20 (Thermoscientific, rabbit polyclonal, 1:400, UK), x40 objective. **C**: Cells with dendritic futures in the interfollicular zone were positive for Iba-1 (Wako, rabbit polyclonal, 1:200, Japan, x40 objective, Inset: Cropped magnified image. These findings supported some cross-reactivity of these antibodies for hedgehog antigens. D: Application of secondary antibody alone did not yield any positive staining (Histofine Max-PO(MULTI), Nichirei, Japan).

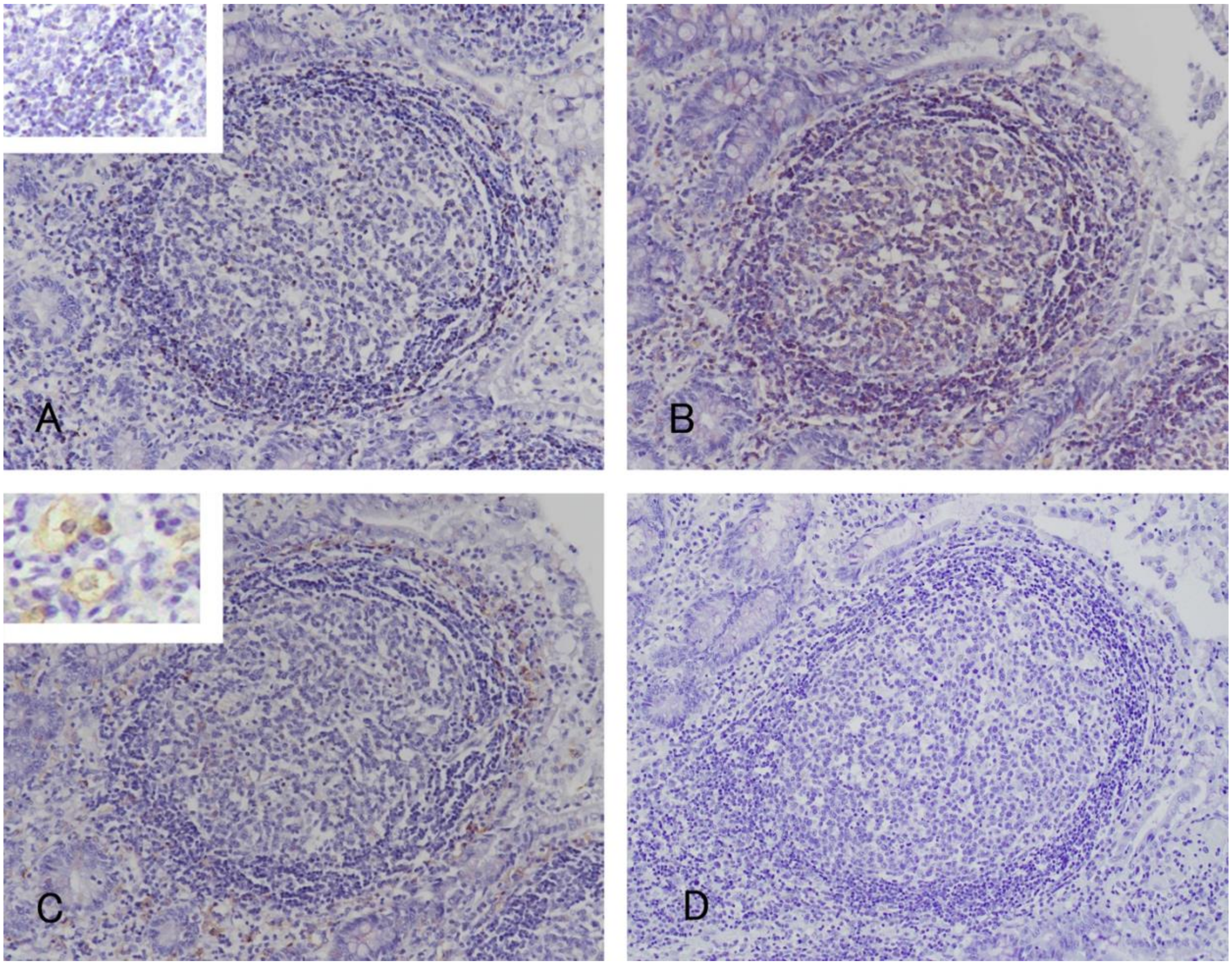

Supplement: Supplementary file 1 [file Data_Sheet_1.PDF]
